# Eighteen Novel Bioactive Peptides from Monkfish (*Lophius litulon*) Swim Bladders: Production, Identification, Antioxidant Activity, and Stability

**DOI:** 10.3390/md21030169

**Published:** 2023-03-07

**Authors:** Yan Sheng, Wan-Yi Wang, Ming-Feng Wu, Yu-Mei Wang, Wang-Yu Zhu, Chang-Feng Chi, Bin Wang

**Affiliations:** 1 Zhejiang Provincial Engineering Technology Research Center of Marine Biomedical Products, School of Food and Pharmacy, Zhejiang Ocean University, Zhoushan 316022, China; 2 National and Provincial Joint Laboratory of Exploration, Utilization of Marine Aquatic Genetic Resources, National Engineering Research Center of Marine Facilities Aquaculture, School of Marine Science and Technology, Zhejiang Ocean University, Zhoushan 316022, China; 3 Cell and Molecular Biology Laboratory, Zhoushan Hospital, Zhoushan 316021, China

**Keywords:** monkfish (*Lophius litulon*), swim bladders, peptide, antioxidant activity, stability

## Abstract

In the study, papain was chosen from five proteases to hydrolyze proteins of monkfish swim bladders for effectively utilizing monkfish (*Lophius litulon*) processing byproducts, and the hydrolysis conditions of papain were optimized as hydrolysis temperature of 65 °C, pH 7.5, enzyme dose 2.5% and time 5 h using single-factor and orthogonal experiments. Eighteen peptides were purified from the swim bladder hydrolysate of monkfish by ultrafiltration and gel permeation chromatography methods and identified as YDYD, QDYD, AGPAS, GPGPHGPSGP, GPK, HRE, GRW, ARW, GPTE, DDGGK, IGPAS, AKPAT, YPAGP, DPT, FPGPT, GPGPT, GPT and DPAGP, respectively. Among eighteen peptides, GRW and ARW showed significant DPPH· scavenging activities with EC_50_ values of 1.053 ± 0.003 and 0.773 ± 0.003 mg/mL, respectively; YDYD, QDYD, GRW, ARW and YPAGP revealed significantly HO· scavenging activities with EC_50_ values of 0.150 ± 0.060, 0.177 ± 0.035, 0.201 ± 0.013, 0.183 ± 0.0016 and 0.190 ± 0.010 mg/mL, respectively; YDYD, QDYD, ARW, DDGGK and YPAGP have significantly $O_{2}^{-}\cdot$ scavenging capability with EC_50_ values of 0.126 ± 0.0005, 0.112 ± 0.0028, 0.127 ± 0.0002, 0.128 ± 0.0018 and 0.107 ± 0.0002 mg/mL, respectively; and YDYD, QDYD and YPAGP showed strong ABTS^+^· scavenging ability with EC_50_ values of 3.197 ± 0.036, 2.337 ± 0.016 and 3.839 ± 0.102 mg/mL, respectively. YDYD, ARW and DDGGK displayed the remarkable ability of lipid peroxidation inhibition and Ferric-reducing antioxidant properties. Moreover, YDYD and ARW can protect Plasmid DNA and HepG2 cells against H_2_O_2_-induced oxidative stress. Furthermore, eighteen isolated peptides had high stability under temperatures ranging from 25–100 °C; YDYD, QDYD, GRW and ARW were more sensitive to alkali treatment, but DDGGK and YPAGP were more sensitive to acid treatment; and YDYD showed strong stability treated with simulated GI digestion. Therefore, the prepared antioxidant peptides, especially YDYD, QDYD, GRW, ARW, DDGGK and YPAGP from monkfish swim bladders could serve as functional components applied in health-promoting products because of their high-antioxidant functions.

## 1. Introduction

Bioactive peptides (BPs) comprise 3–30 amino acid (AA) residues with molecular weights (MWs) ranging from 500 to 1850 Da and are generated from diversified protein resources by enzymatic hydrolysis, chemical degradation and microbial fermentation methods [1,2,3]. In addition to their widely accepted nutritive value, BPs have also been proven to have important applications in promoting human health due to their significant physiological and pharmacological functions [4,5,6]. Then, BPs draw great interest from consumers and have been applied in a wide variety of products, especially functional food, daily cosmetics, medicinal/pharmaceutical products, and nutritional supplements [7,8,9].

Reactive oxygen species (ROS) are the metabolites of the respiration process and take on an important role in the physiological and biochemical reactions of organisms [10]. Normally, the body’s oxidative and antioxidant defense systems are in balance, and ROS performed many specific roles including serving as cell-growth factors and regulators of signals between cells [11,12]. In contrast, an overwhelming amount of ROS will be produced in the body under adverse exogenous environmental stress or during disease episodes. Even worse, overexpression and residual ROS can disrupt the balance, lead to oxidative stress and cause damage to cellular macromolecules as confirmed by nitrotyrosine, lipid peroxidation, and DNA/RNA and protein oxidation [13,14,15]. Moreover, numerous examples of literature have reported that oxidative stress was a significant contributor to the emergence and progression of various chronic non-communicable diseases (NCD) such as neurological dysfunction, hypertension, cardiovascular, atherosclerosis, diabetes, cancer and auto-immune disease [16,17,18,19,20,21]. According to data released by the World Health Organization (WHO) in 2019, NCD is one of the top 10 threats to global health and led to 70% of deaths all over the world, and oxidative damage is regarded as a major cause of NCD [22]. Antioxidants can clear away excessive ROS and preserve the organisms from oxidative injury and its adverse reactions [23,24]. Therefore, several chemical synthetic antioxidants, such as butylated hydroxytoluene (BHT), butylated hydroxyanisole (BHA) and tertbutylhydroquinone (TBHQ), present powerful antioxidant functions, but they show some alarming toxic effects on body health, such as DNA damage, hepatotoxicity and carcinogenesis [25,26]. For example: Achar et al. reported that toxicity assessment on BHT revealed carcinogenic, teratogenic and mutagenic effects in animal models [27]; BHT could induce hyperactivity and alters dopamine-related gene expression in larval zebrafish (*Danio rerio*) [28]; TBHQ could cause considerable DNA fragmentation, necrosis and apoptosis [29]. Thus, actions have been taken to restrict the usage of synthetic antioxidants in consideration of their potential toxicity by consumers [30,31]. Meanwhile, the search for safe and efficient natural antioxidants has caused widespread concern.

Presently, some natural antioxidants, including flavone, quinonoid, sappanone, curcumin, phenol, melatonin and peptides have been rapidly screened and produced using animal, plant and microorganism resources [32,33,34]. Among those bioactive substances, antioxidant peptides (APs) are gained scientific interest for their potential advantages in preventing and treating NCD [8,35,36]. For example, VIAPW and IRWWW isolated from miiuy croaker (*Miichthys miiuy*) muscle could regulate the AMPK pathway to control the expression levels of some proteolytic enzymes in lipid synthesis and oxidation process and further decrease the intracellular levels of TG and TC [37]. Tripeptide WLP derived from sea squirt (*Halocynthia roretzi*) could relieve neurodegenerative disorders related to oxidative stress through attenuating cell apoptosis, decreasing ROS levels, and remarkably enhancing GSH-Px activity [38]. Oyster (*Crassostrea giga)* peptides (MW < 3500 Da) displayed prominent liver-protection ability in mice models of alcohol-caused hepatopathy via regulating the Nrf2-ARE pathway to heighten the antioxidant capability and inactivating NF-κB pathway to control the inflammatory reaction [39]. DREL from Jiuzao could activate these signaling pathways, including Nrf2/Keap1, p38/PI3K and MafK, to improve the antioxidative and anti-inflammatory ability in the AAPH-induced Sprague Dawley (SD) rat [40]. Consequently, APs have great potential in the functionality of food, nutraceutical supplements and pharmaceutical products for preventing and treating chronic and degenerative diseases.

Monkfish (*Lophius litulon*) belongs to the members of the genus *Lophiidae*, lives at the bottom of the offshore sea, and is found mainly in the Indian, Atlantic and Pacific Oceans [41]. Presently, BPs have been produced from monkfish muscle and its processing by-products and showed significant bioactivities. For example, peptides fraction (MW < 1 kDa) from monkfish muscle could improve the antioxidant capacity of the liver to alleviate non-alcoholic fatty liver disease (NAFLD) progression mainly through modulating the intestinal flora and AMPK and Nrf2 pathways [42,43,44]. APs prepared from monkfish muscle hydrolysate, including EWPAQ, FLHRP, LMGQW, EDIVCW, MEPVW and YWDAW, could concentration-dependently scavenge free radicals and control lipid peroxidation [18,41]. Low MW peptides from monkfish roes could enhance the immune regulatory effect in immunosuppressive mice by activating the signaling pathways of NF-κB/MAPK in spleen tissues [45]. Collagen peptides from monkfish skin could protect mice against the kidney damage induced by a high-fat diet by regulating the signaling pathways of Nrf2/NLRP3 [46]. However, there is no study on BPs from monkfish swim bladders. Therefore, the objectives of the study were to produce and characterize APs from the swim bladder hydrolysate of monkfish for efficient utilization of monkfish processing byproducts. Moreover, we comprehensively determined and evaluated the antioxidant capability and stability of eighteen prepared APs (MSP1 to MSP18).

## 2. Results

### 2.1. Preparation of Protein Hydrolysate of Monkfish Swim Bladders (MSBH)

#### 2.1.1. Screening of Enzyme Species

**Figure 1:**
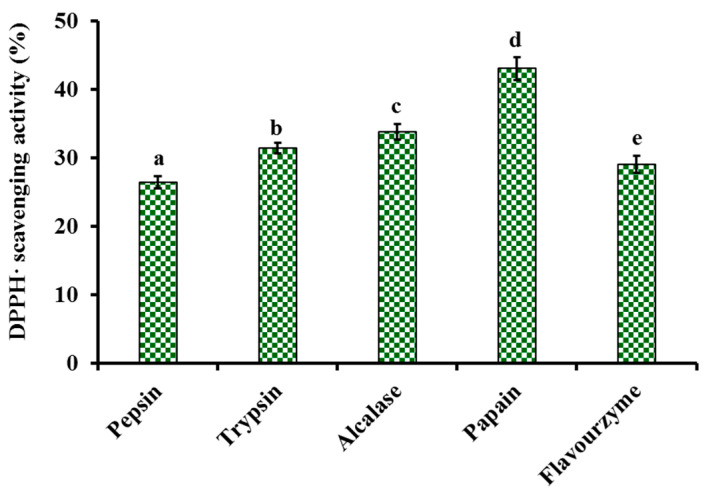
Influences of enzyme species on DPPH· scavenging activity of monkfish (*L. litulon*) swim bladder hydrolysates at 5.0 mg/mL. All data are presented as the mean ± SD of triplicate results. ^a–e^ Values with the same letters indicate no significant difference (*p* > 0.05).

To more thoroughly hydrolyze the protein of monkfish swim bladders, five kinds of proteases, including pepsin, trypsin, alcalase, papain and flavourzyme, were selected to evaluate their hydrolyzing capacity (Figure 1). At 5.0 mg/mL, the DPPH· scavenging ratio of produced hydrolysate by papain was 43.09 ± 1.69%, and the ratio was significantly higher than those of produced hydrolysates using pepsin (26.48 ± 0.87%), trypsin (31.47 ± 0.77%), alcalase (33.84 ± 1.14%) and flavourzyme (29.06 ± 1.2%), respectively (*p* < 0.05). Therefore, papain was chosen for the preparation of hydrolysates from monkfish swim bladders.

#### 2.1.2. Optimization of Hydrolysis Conditions of Papain

**Figure 2:**
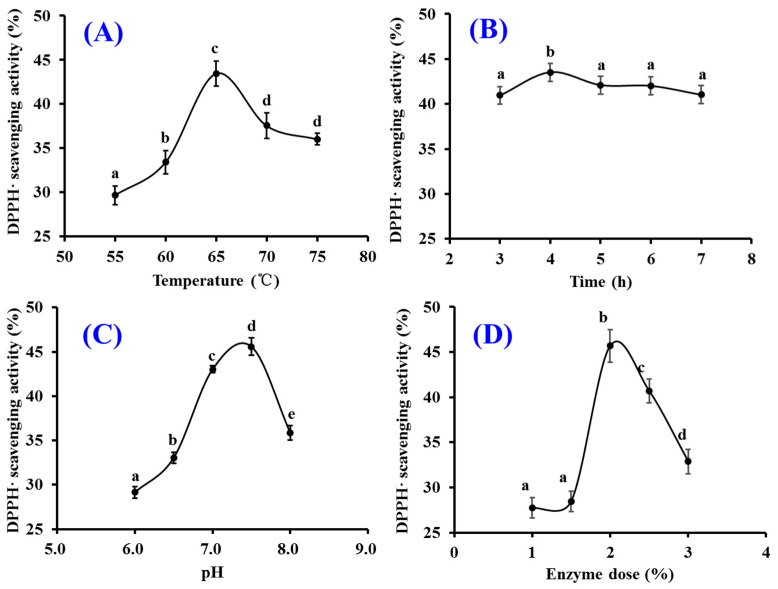
Effects of hydrolysis temperature (**A**), time (**B**), pH (**C**) and enzyme dose (**D**) of papain on DPPH· scavenging activity of monkfish swim bladder hydrolysates at 5.0 mg/mL. All data are presented as the mean ± SD of triplicate results. ^a–e^ Values with the same letters indicate no significant difference (*p* > 0.05).

The hydrolysis conditions of papain including hydrolysis temperature (A), time (B), pH (C) and enzyme dose (D) on the influence of the radical scavenging activity of monkfish swim bladder hydrolysates were optimized using the single-factor experiment (Figure 2). Figure 2A depicted that hydrolysis temperature significantly affected the radical scavenging capability of swim bladder hydrolysates, and the DPPH· scavenging rate (43.47 ± 1.41%) of prepared hydrolysate at 65 °C was significantly higher than those of prepared hydrolysates at other temperature (*p* < 0.05). The DPPH· clearance rate of prepared hydrolysate showed a decreasing trend when the temperature was lower or higher than 65 °C. Figure 2B displayed that DPPH· clearance rates of swim bladder hydrolysates increased gradually with the prolongation of hydrolysis time and reached the maximum value (43.53 ± 0.96%) at 4 h. Prolongation of hydrolysis time caused a persistent decrease in the activity of hydrolysates. Figure 2C indicated that DPPH· scavenging rate reached the maximum value (45.58 ± 0.15%) at pH 7.5. The inappropriate pH value of enzymolysis solution can affect the binding of enzyme and substrate by destroying the active center or spatial structure of papain, which further reduced its catalytic activity. Figure 2D displayed that the activity curve showed a trend of rapid rise at the enzyme dose was 1–2.0% and a slow decline when the dose was higher than 2.0%. The highest DPPH· clearance rate was 45.68 ± 0.38% when the enzyme followed by was 2.0%. According to the above experimental results, the ranges of hydrolytic conditions for papain were narrowed down to 60–70°C, 3–5 h, 7.0–8.0, 1.5–2.5% and 3–5 h for hydrolysis temperature, time, pH and enzyme dose, respectively.

The orthogonal test L_9_(3)^4^ was designed for optimizing the hydrolysis conditions of papain (Table 1). Following the *R* values, the conditions interfering with the antioxidant activity of monkfish swim bladder hydrolysates were listed in decreasing order: C (enzyme dose) > B (pH) > D (hydrolysis time) > A (hydrolysis temperature). The enzyme dose was recognized as the most important condition influencing the antioxidant activity of swim bladder hydrolysates. By verification experiments, the maximum DPPH· scavenging ratio of monkfish swim bladder hydrolysate was 47.13 ± 1.15% at 5.0 mg/mL on the optimal enzymolysis level of A2B2C3D3, that is, the optimum conditions of papain for producing monkfish swim bladder hydrolysate were hydrolysis temperature 65 °C, pH 7.5, enzyme dose 2.5% and time 5 h. In addition, the monkfish swim bladder hydrolysate produced under the optimal conditions of papain was named MSBH.

### 2.2. Preparation of APs from MSBH

#### 2.2.1. Ultrafiltration

**Figure 3:**
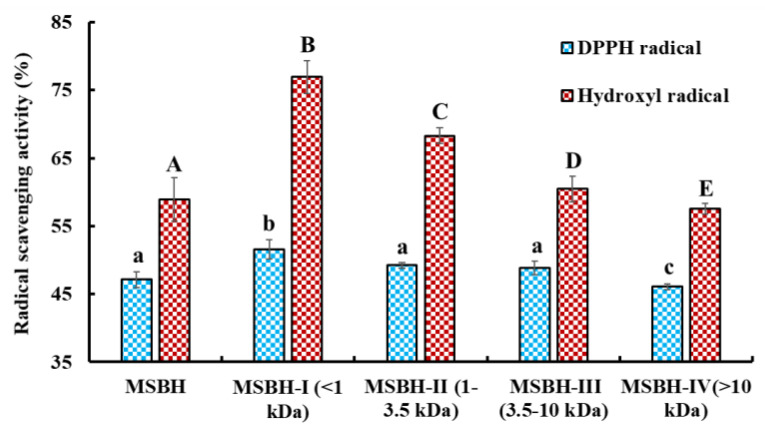
Radical scavenging activity of the monkfish swim bladder hydrolysate (MSBH) and its four fractions (MSBH-I to MSBH-IV). All data are presented as the mean ± SD of triplicate results. ^a–c^ or ^A–E^ Values with the same letters indicate no significant difference (*p* > 0.05).

The radical scavenging rates of MSBH and its four ultrafiltration fractions (MSBH-I, MW < 1 kDa; MSBH-II, 1 kDa < MW < 3.5 kDa; MSBH-III, 3.5 kDa < MW < 10 kDa; MSBH-IV, MW > 10 kDa) at 5.0 mg/mL were measured (Figure 3). The data manifested that DPPH· and HO· scavenging rates of MSBH-I were 51.57 ± 1.45% and 76.96 ± 2.40%. The rates of MSBH-I were significantly greater than those of MSBH (47.13 ± 1.18% and 58.91 ± 3.17%), MSBH-II (49.23 ± 0.42% and 68.29 ± 1.141%), MSBH-III (48.86 ± 1.00% and 60.45 ± 1.92%) and MSBH-IV (46.11 ± 0.42% and 57.54 ± 0.81%) (*p* < 0.05). Then, MSBH-I was chosen for subsequent separation.

#### 2.2.2. Chromatography of MSBH

**Figure 4:**
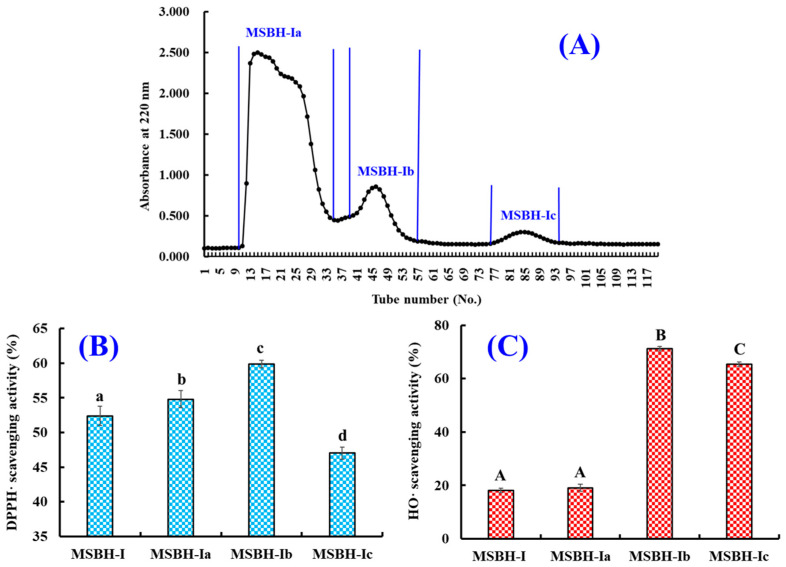
Chromatographic diagram of MSBH-I isolated by Sephadex G-15 and the radical scavenging activity of prepared subfractions (MSBH-Ia, MSBH-Ib and MSBH-Ic) from MSBH-I. (**A**) Chromatographic diagram of MSBH-I; (**B**) DPPH· scavenging activity; (**C**) HO· scavenging activity. All data are presented as the mean ± SD of triplicate results. ^a–d^ or ^A–C^ Values with the same letters indicate no significant difference (*p* > 0.05).

Using the Sephadex G-15 column, MSBH was fractionated into three subfractions (MSBH-Ia, MSBH-Ib and MSBH-Ic) according to the chromatographic diagram at 220 nm (Figure 4A). The DPPH· scavenging abilities of MSBH-Ib were 59.91 ± 0.55% at 5.0 mg/mL, which were significantly higher than those of MSBH-I (52.41 ± 1.42%), MSBH-Ia (59.91 ± 0.55%) and MSBH-Ic (47.04 ± 0.87%) (*p* < 0.05). At 1.0 mg/mL, HO· scavenging abilities of MSBH-Ib were 71.19 ± 0.85%, which were significantly higher than those of MSBH-I (18.04 ± 0.83%), MSBH-Ia (19.07 ± 1.33%) and MSBH-Ic (65.33 ± 0.79%), respectively (*p* < 0.05).

**Figure 5:**
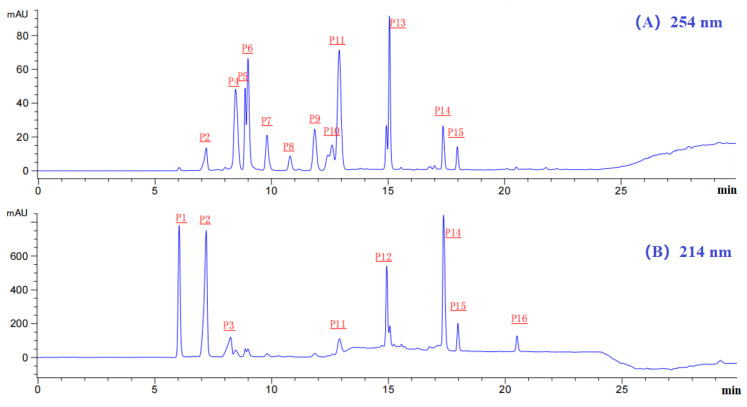
Chromatographic diagrams of MSBH-Ib by RP-HPLC at 254 nm (**A**) and 214 nm (**B**).

Finally, MSBH-Ib with high DPPH· and HO· scavenging abilities was isolated using RP-HPLC. By the chromatographic diagrams of MSBH-Ib at 214 and 254 nm (Figure 5), sixteen chromatographic peaks (P1–P16) with retention time (RT, min) of 6.08 (P1), 7.14 (P2), 8.22 (P3), 8.30 (P4), 8.93 (P5), 9.02 (P6), 9.87 (P7), 10.89 (P8), 10.91 (P9), 12.60 (P10), 13.92 (P11), 14.93 (P12), 15.02 (P13), 17.18 (P14), 17.99 (P15) and 20.50 min (P16) were collected lyophilized (Table 2). Then, sixteen components (P1–P16) separated by RP-HPLC were enriched for structure identification.

### 2.3. Determination of the AA Sequences and MWs of Eighteen Isolated APs (MSP1-MSP18)

The MWs and sequences of APs in HPLC chromatographic peaks (P1–P16) were determined by protein sequencer and ESI-MS (Table 2). Peaks of P1 and P10 contained two peptides. Therefore, eighteen APs (MSP1-MSP18) from chromatographic peaks of P1–P16 were identified as Tyr-Asp-Tyr-Asp (YDYD, MSP1), Gln-Asp-Tyr-Asp (QDYD, MSP2), Ala-Gly-Pro-Ala-Ser (AGPAS, MSP3), Gly-Pro-Gly-Pro-His-Gly-Pro-Ser-Gly-Pro (GPGPHGPSGP, MSP4), Gly-Pro-Lys (GPK, MSP5), His-Arg-Glu (HRE, MSP6), Gly-Arg-Trp (GRW, MSP7), Ala-Arg-Trp (ARW, MSP8), Gly-Pro-Thr-Glu (GPTE, MSP9), Asp-Asp-Gly-Gly-Lys (DDGGK, MSP10), Ile-Gly-Pro-Ala-Ser (IGPAS, MSP11), Ala-Lys-Pro-Ala-Thr (AKPAT, MSP12), Tyr-Pro-Ala-Gly-Pro (YPAGP, MSP13), Asp-Pro-Thr (DPT, MSP14), Phe-Pro-Gly-Pro-Thr (FPGPT, MSP15), Gly-Pro-Gly-Pro-Thr (GPGPT, MSP16), Gly-Pro-Thr (GPT, MSP17) and Asp-Pro-Ala-Gly-Pro (DPAGP, MSP18), respectively. In addition, the MWs of eighteen APs (MSP1-MSP18) were determined as 574.54, 539.49, 401.41, 858.89, 300.35, 440.45, 417.46, 431.49, 402.40, 490.47, 443.49, 486.56, 503.54, 331.32, 517.57, 427.45, 273.29 and 455.46 Da, respectively, which were quite consistent with their theoretical MWs (Table 2).

### 2.4. Antioxidant Activity of MSP1 to MSP18

#### 2.4.1. Radical Scavenging Activity of Eighteen Isolated APs (MSP1-MSP18)

**Figure 6:**
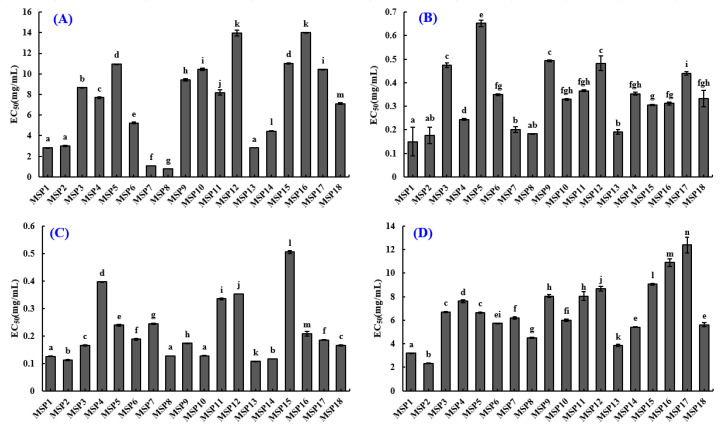
Radical scavenging activity of eighteen isolated APs (MSP1-MSP18). (**A**) EC_50_ values of peptides (MSP1-MSP18) on DPPH·; (**B**) EC_50_ values of peptides (MSP1-MSP18) on HO·; (**C**) EC_50_ values of peptides (MSP1-MSP18) on $O_{2}^{-}\cdot$; (**D**) EC_50_ values of peptides (MSP1-MSP18) on ABTS^+^·. All data are presented as the mean ± SD of triplicate results. ^a–n^ Values with the same letters indicate no significant difference (*p* > 0.05).

Figure 6A and Table 3 showed that the EC_50_ values of MSP7 and MSP8 on DPPH· were 1.053 ± 0.003 and 0.773 ± 0.003 mg/mL, which were significantly lower than those of the other sixteen isolated APs (*p* < 0.05).

The data in Figure 6B and Table 3 depicted that MSP1 had the minimum EC_50_ (0.150 ± 0.060 mg/mL) on HO· among eighteen APs (MSP1 to MSP18) and followed by MSP2 (0.177 ± 0.035 mg/mL), MSP7 (0.201 ± 0.013 mg/mL), MSP8 (0.183 ± 0.0016 mg/mL) and MSP13 (0.190 ± 0.010 mg/mL).

Figure 6C and Table 3 showed MSP1, MSP2, MSP8, MSP10 and MSP13 have significant $O_{2}^{-}\cdot$ scavenging capability with EC_50_ values of 0.126 ± 0.0005, 0.112 ± 0.0028, 0.127 ± 0.0002, 0.128 ± 0.0018 and 0.107 ± 0.0002 mg/mL, respectively. EC_50_ values of MSP1, MSP2, MSP8, MSP10 and MSP13 on $O_{2}^{-}\cdot$ were significantly lower than those of the other thirteen isolated peptides.

Figure 6D and Table 3 indicated that eighteen isolated peptides (MSP1-MSP18) showed relatively weak ABTS^+^· scavenging ability. The EC_50_ values of MSP1, MSP2 and MSP13 were 3.197 ± 0.036, 2.337 ± 0.016 and 3.839 ± 0.102 mg/mL, respectively, which were remarkably lower than those of the other fifteen isolated peptides.

In addition, we comprehensively considered the radical scavenging activities of eighteen isolated APs (MSP1-MSP18), and finally selected MSP1, MSP2, MSP7, MSP8, MSP10 and MSP13 for subsequent bioactive and stability experiments.

#### 2.4.2. Lipid Peroxidation Inhibition Ability

**Figure 7:**
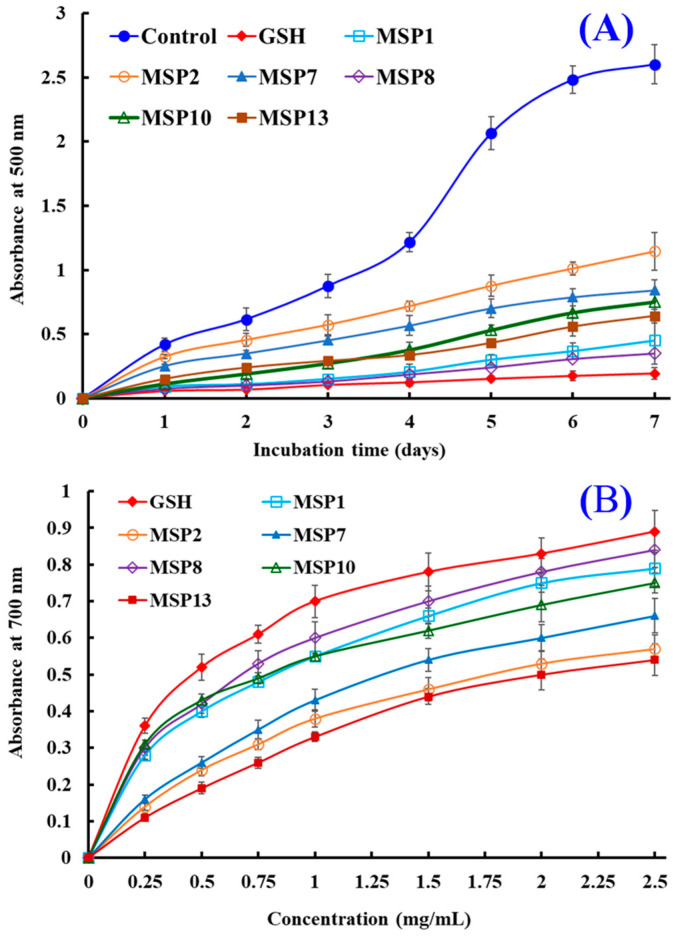
The lipid peroxidation inhibition ability (**A**) and ferric reducing antioxidant power (FRAP) (**B**) of MSP1, MSP2, MSP7, MSP8, MSP10 and MSP13. All data are presented as the mean ± SD of triplicate results.

In organic tissues, lipid peroxidation is generally described as a process in which oxidants attack lipids containing polyunsaturated fatty acids (PUFAs), which has an important role in human health because lipid peroxidation products, including MDA and 4-HNE, play a vital cytotoxic role in promoting cell death and controlling gene expression [47]. Therefore, the assay was applied to comprehensively evaluate the activities of MSP1, MSP2, MSP7, MSP8, MSP10 and MSP13 (Figure 7A). In the linoleic acid emulsion system, the values at 500 nm of MSP1, MSP2, MSP7, MSP8, MSP10 and MSP13 were significantly smaller than that of blank control (without peptide and GSH) during 7 days. The finding demonstrated that MSP1, MSP2, MSP7, MSP8, MSP10 and MSP13 could effectively inhibit the reaction rate and efficiency of lipid peroxidation in the experimental system by reacting with H_2_O_2_. Moreover, the MSP8 showed a similar inhibiting capability to that of GSH, followed by MSP1, MSP10 and MSP13.

#### 2.4.3. Ferric Reducing Antioxidant Power (FRAP)

The FRAP assay reflects the ability of compounds that serve as electron donors to decrease the oxidized intermediates in the lipid peroxidation process, and it has been used as a preferred method to evaluate the “total antioxidant content” of functional molecules [48,49]. As shown in Figure 6B, MSP8 showed a higher ability to convert Fe^3+^/ferricyanide complex into Fe^2+^ form than the other five determined peptides, followed by MSP1, MSP10 and MSP7. However, the reducing power of MSP1, MSP2, MSP7, MSP8, MSP10 and MSP13 was lower than that of glutathione (GSH).

#### 2.4.4. Protective Effect on H_2_O_2_-Damaged Plasmid DNA

**Figure 8:**
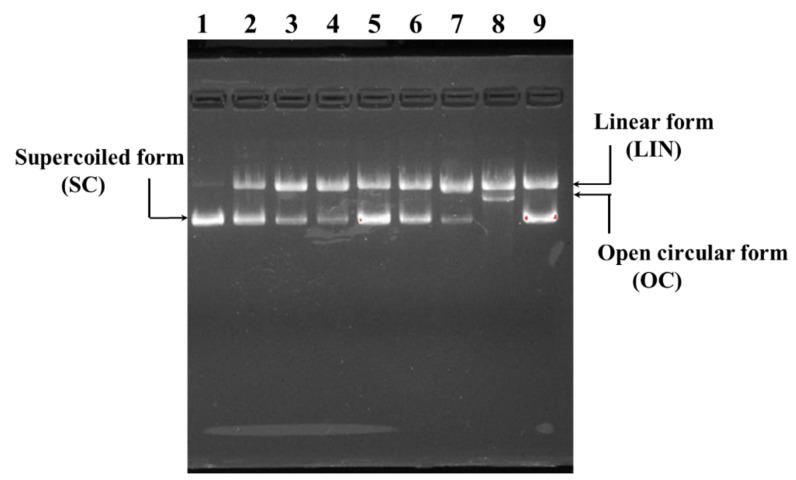
The protective effects of MSP1, MSP2, MSP7, MSP8, MSP10 and MSP13 on the H_2_O_2_-damaged plasmid DNA (pBR322DNA). Lane 1: native pBR322DNA; Lane 2–9, DNA + FeSO_4_ + H_2_O_2_ + MSP1, MSP2, MSP7, MSP8, MSP10, MSP13, H_2_O and GSH, respectively.

Figure 8 indicated the protective ability of MSP1, MSP2, MSP7, MSP8, MSP10 and MSP13 on pBR322DNA against H_2_O_2_ damage. Compared with the supercoiled (SC) form of pBR322DNA with two phosphodiester chains (Figure 8, lane 1), the phosphodiester chains of plasmid DNA were clipped by the HO· and formed a linear (LIN) or open circular (OC) structure in the model group (Figure 8, lane 8). Nevertheless, more SC structures were preserved in the pBR322DNA of MSP1, MSP2, MSP7, MSP8, MSP10 and MSP13 groups (Figure 8, lane 2–7), which indicated that MSP1, MSP2, MSP7, MSP8, MSP10 and MSP13 could protect plasmid DNA (pBR322DNA) against the damage by HO·. In addition, the protective function of MSP1 and MSP8 was similar to that of GSH and significantly stronger than those of MSP2, MSP7, MSP10 and MSP13.

#### 2.4.5. Cytoprotective Function on H_2_O_2_-Damaged HepG2 Cells

**Figure 9:**
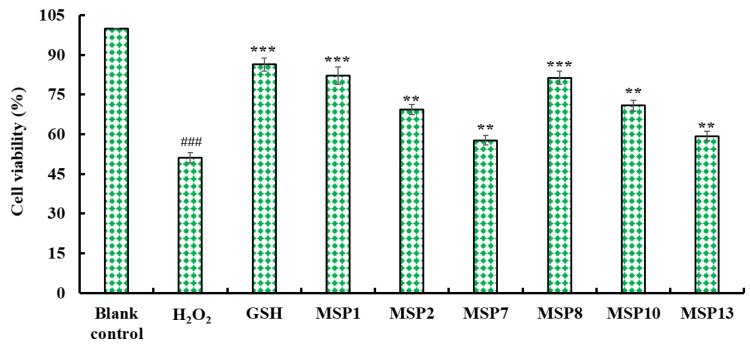
Cytoprotective effects of MSP1, MSP2, MSP7, MSP8, MSP10 and MSP13 on the H_2_O_2_-induced HepG2 cells at 200 μmol/L. GSH served as the positive control. All data are presented as the mean ± SD of triplicate results. ^###^ *p* < 0.001 vs. blank control group; *** *p* < 0.001 and ** *p* < 0.01 vs. H_2_O_2_-induced model group.

Figure 9 indicated that MSP1, MSP2, MSP7, MSP8, MSP10 and MSP13 could increase the viability of H_2_O_2_-injuried HepG2 cells at 200 μmol/L. In addition, the viability of H_2_O_2_-injured HepG2 cells in MSP1 and MSP8 groups was 82.14 ± 3.28% and 81.32 ± 2.45%, which were significantly higher than that of the model group (51.08 ± 1.97%). Therefore, MSP1, MSP2, MSP7, MSP8, MSP10 and MSP13 presented cytoprotective function to H_2_O_2_-injuried HepG2 cells by increasing the cell viability.

### 2.5. Stability of MSP1, MSP2, MSP7, MSP8, MSP10 and MSP13

#### 2.5.1. pH Stability of MSP1, MSP2, MSP7, MSP8, MSP10 and MSP13

**Figure 10:**
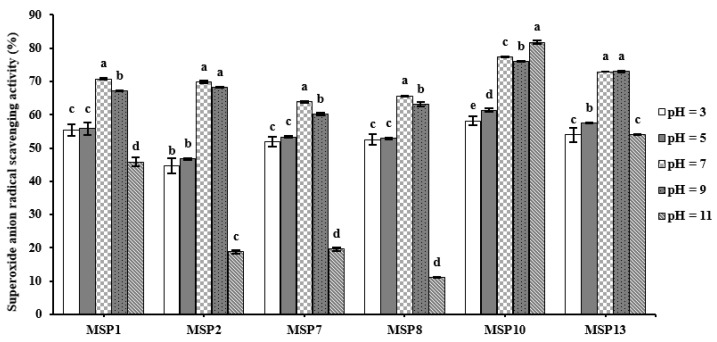
$O_{2}^{-}\cdot$ scavenging activity of MSP1, MSP2, MSP7, MSP8, MSP10 and MSP13 subjected to different pH treatments. All data are presented as the mean ± SD of triplicate results. ^a–e^ values with the same letters indicate no significant difference of the same peptide (*p* > 0.05).

Figure 10 and Table 4 indicated that MSP1, MSP2, MSP7, MSP8, MSP10 and MSP13 showed significant differences in tolerance to acid and alkali treatment (pH 3 to 11). MSP1, MSP2, MSP7 and MSP8 kept the highest $O_{2}^{-}\cdot$ scavenging activity at pH 7.0, but MSP10 and MSP13 kept the highest activity at pH 11.0 and 9.0, respectively. In addition, MSP1, MSP2, MSP7 and MSP8 were more sensitive to alkali treatment because their $O_{2}^{-}\cdot$ scavenging rates dropped by 35.26%, 73.07%, 69.19% and 83.05%, respectively, at pH 11.0. Conversely, MSP10 and MSP13 were more sensitive to acid treatment because their $O_{2}^{-}\cdot$ scavenging rates dropped by 26.26% and 33.05% at pH 3.0. Those data suggested that MSP1, MSP2, MSP7 and MSP8 are appropriate for the application to products in a neutral environment, but MSP10 and MSP13 are appropriate for the application to products in an alkali environment.

#### 2.5.2. Thermal Stability of MSP1, MSP2, MSP7, MSP8, MSP10 and MSP13

**Figure 11:**
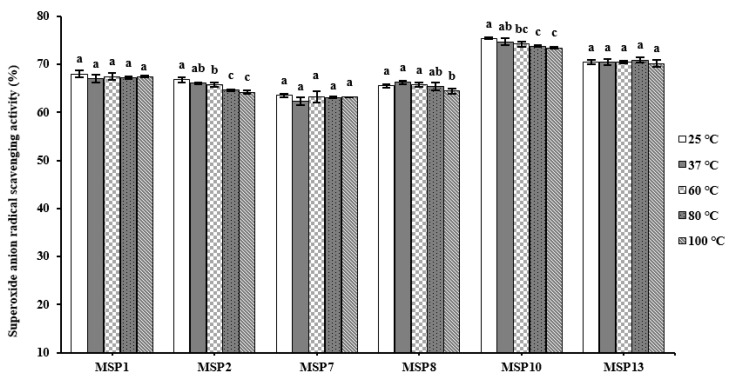
$O_{2}^{-}\cdot$ scavenging activity of MSP1, MSP2, MSP7, MSP8, MSP10 and MSP13 subjected to different thermal treatments. All data are presented as the mean ± SD of triplicate results. ^a–c^ Values with the same letters indicate no significant difference of same peptide (*p* > 0.05).

The $O_{2}^{-}\cdot$ scavenging activity of MSP1, MSP2, MSP7, MSP8, MSP10 and MSP13 treated using different temperatures (25, 37, 60, 80 and 100 °C) was present in Figure 11, and the results manifested that six APs have high-temperature tolerance. Compared with MSP1, MSP7, MSP8 and MSP13, MSP2 and MSP10 were relatively affected by high temperature (100 °C), and their $O_{2}^{-}\cdot$ scavenging activity, respectively, decreased by 3.84% and 2.60%. Those data suggested that MSP1, MSP2, MSP7, MSP8, MSP10 and MSP13 are appropriate for the application to products treated by high temperatures because of their high-temperature tolerance.

#### 2.5.3. Stability of MSP1, MSP2, MSP7, MSP8, MSP10 and MSP13 Subjected to Simulated Gastrointestinal (GI) Digestion

**Figure 12:**
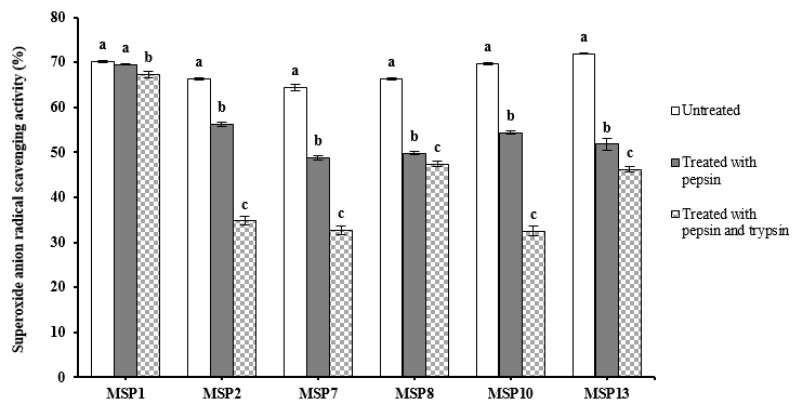
$O_{2}^{-}\cdot$ scavenging activity of MSP1, MSP2, MSP7, MSP8, MSP10 and MSP13 subjected to simulated GI digestion treatments. All data are presented as the mean ± SD of triplicate results. ^a–c^ Values with the same letters indicate no significant difference in the same sample (*p* > 0.05).

Figure 12 presented the effects of simulated GI digestion on the antioxidant ability of MSP1, MSP2, MSP7, MSP8, MSP10 and MSP13, and the results indicated that $O_{2}^{-}\cdot$ scavenging activities of MSP1, MSP2, MSP7, MSP8, MSP10 and MSP13 decreased gradually when they were treated with pepsin and trypsin in turn. Among six peptides, the $O_{2}^{-}\cdot$ scavenging rates of MSP2, MSP7 and MSP10 showed the most seriously affected after simulated GI digestion and decreased by 47.59%, 49.19% and 53.33%, respectively; the $O_{2}^{-}\cdot$ scavenging rates of MSP8 and MSP13 were also affected and decreased by 28.62% and 35.83%, respectively(Table 5). However, MSP1 showed good tolerance and its $O_{2}^{-}\cdot$ scavenging rate only decreased by 4.1% (Table 6). Those results suggested that MSP1 showed high stability when it was treated with simulated GI digestion.

## 3. Discussion

### 3.1. Preparation of APs from MSBH

BPs are encapsulated in the sequence of proteins and keep inactive form and can be released by a variety of hydrolysis pathways [28]. In comparison with chemical and micro-biological degradation, protease degradation is known as an effective, safe and rational method for the production of protein hydrolysates because of the high controllability and reproducibility of the enzymatic process, the mild and safe conditions of enzymatic protein digestion and the absence of side reactions in the enzymatic reaction. Then, protease degradation is more widely used in the food and pharmaceutical industries [3,50,51]. Therefore, proteases including papain, alcalase, pepsin, flavourzyme, Protamex^®^, trypsin and their combinations are frequently used to manufacture BPs from marine organisms and their by-products [9,52,53]. In addition, many enzymes have specific cleavage sites (papain: Arg-, Lys- and Phe-; alcalase: Ala-, Leu-, Val-, Tyr-, Phe- and Try-; trypsin: Arg- and Lys-; pepsin: Phe- and Leu-), and different cleavage sites will have a certain effect on the activity of hydrolysates [54,55]. Ktari et al. found that hydrolysates from cuttlefish (*Sepia officinalis*) by-products obtained by alcalase and sardinelle crude enzyme exhibited the strongest activity among eight hydrolysates [52]. Alcalase hydrolysate of Antarctic Krill had the highest radical scavenging ability among the five hydrolysates [56]. Nangnoi strain hydrolysate generated by alcalase showed the highest activities among the three hydrolysates [57]. In the study, MSBH produced by papain shows the highest activity further proving this conclusion that specificity and conditions of proteases are the key factors for the generation of APs.

The hydrolysate profile is also influenced by enzyme concentration, digestion time, digestion temperature, ambient pH and other factors. At the optimum pH and temperature, the cleavage rate of protease is accelerated and non-specific digestion sites are less likely to occur. In addition, insufficient enzymatic digestion may occur with too short a digestion time and too low an enzyme concentration [3,12]. Jang et al. found that the optimal hydrolysis conditions for alcalase 2.4 L were pH 6.0, temperature 70 °C, enzyme concentration 5% (*w*/*w*), and hydrolysis time 3 h. The optimal hydrolysis conditions for Collupulin MG were pH 9.0, temperature 60 °C, enzyme dose 5% (*w*/*w*), and the DPPH· radical scavenging activity of the two hydrolysates under optimal conditions was 60.04 and 79.65%, respectively [58]. In the study, the optimum conditions of enzymatic hydrolysis of papain were temperature 65 °C, pH 7.5, enzyme dose 2.5%, enzymatic hydrolysis time 2 h and the DPPH· scavenging rate of enzymatic hydrolysis product was 47.13 ± 1.15%. It was further proved that the enzymatic hydrolysis condition of papain significantly influenced the generation of APs.

Protein hydrolysates consist of peptides with different chain lengths, and MW is also a key factor affecting the separation and production of BPs [28,59]. In consequence, membrane ultrafiltration and gel permeation chromatography become the most popular methods for peptide isolation from different hydrolysates, such as salmon byproduct [32], Antarctic Krill [56], miiuy croaker muscle [49], Skipjack tuna (*K. pelamis*) bone [51], seahorse (*Hippocampus abdominalis*) [60], oyster (*C. giga*) [61] and *E. cottonii* [62]. Refering to the existing literature, we designed the separation method and eighteen APs (MSP1-MSP18) were prepared from MSBH using radical scavenging activity as the screening index. Among eighteen peptides (MSP1-MSP18), MSP1, MSP2, MSP7, MSP8, MSP10 and MSP13 presented remarkable antioxidant activity.

### 3.2. Antioxidant Activity of MSP1, MSP2, MSP7, MSP8, MSP10 and MSP13

DPPH· is a very stable radical and is commonly used as a substrate to assess antioxidant activity. MSP7 and MSP8 have a strong hydrogen donating capacity and can reduce stable DPPH·. A review of the literature found that the EC_50_ values of MSP7 and MSP8 on DPPH· were lower than those of marine APs from scalloped hammerhead sharks (*Sphyrna lewini*) muscle (WDR: 3.63 mg/mL; SAP: 3.06 mg/mL; PYFK: 4.11 mg/mL) [63,64], seaweed (*Eucheuma cottonii*) (YSKT: 1.71 mg/mL; FYKA: 2.56 mg/mL) [31], tuna (*Katsuwonus pelamis*) cardiac arterial bulbs (GEQSN: 2.054 mg/mL; GEGQR: 2.257 mg/mL) [50] and Spanish mackerel (*Scomberomorous niphonius*) skin (GPTGE: 1.42 mg/mL; PYGAKG: 3.02 mg/mL) [62]. However, the EC_50_ values of MSP7 and MSP8 on DPPH· were higher than those of marine APs from Spanish mackerel skin (YGPM: 0.72 mg/mL) [62], monkfish muscle (EDIVCW: 0.39 mg/mL; YWDAW: 0.51 mg/mL) [18] and tuna roes (AEM: 0.250 mg/mL; YVM: 0.288 mg/mL) [65]. This finding suggests that MSP7 and MSP8 can convert DPPH· to less harmful or non-harmful products, breaking the free radical chain reaction.

HO· is a kind of strong free radical, which can not only cause lipid peroxidation in vivo but also attack polysaccharides, nucleic acids and other macromolecules, causing serious damage to cells. MSP1, MSP2, MSP7, MSP8 and MSP13 have strong HO· scavenging ability and their EC_50_ values were lower than those of marine APs from tuna roes (AEM: 0.456 mg/mL; YVM: 0.413 mg/mL) [65], monkfish muscle (EDIVCW: 0.61 mg/mL; YWDAW: 0.32 mg/mL) [18] and skin (GPY: 3.22 mg/mL; YGPM: 0.88 mg/mL) [62], Antarctic krill (*Euphausia superba*) (VEKT: 1.53 mg/mL; IDSQ: 2.57 mg/mL) [29], loach (*Misgurnus anguillicaudatus*) meat (PSYV: 2.64 mg/mL) [66] and grass carp (*Ctenopharyngodon idella*) skin (VGGRP: 2.06 mg/mL) [67]. This finding suggests that MSP1, MSP2, MSP7, MSP8 and MSP13 have a strong ability to scavenge HO· and can reduce the damage to the body caused by HO·.

$O_{2}^{-}\cdot$ reacts with metal ions in the Fenton reaction to form HO· that triggers lipid peroxidation and causes oxidative damage to cells. MSP1, MSP2, MSP8, MSP10 and MSP13 have a strong ability to scavenge $O_{2}^{-}\cdot$. A review of the literature revealed that the EC_50_ values of MSP1, MSP2, MSP8, MSP10 and MSP13 on $O_{2}^{-}\cdot$ were significantly lower than those of marine APs from monkfish muscle (EDIVCW: 0.76 mg/mL; YWDAW: 0.48 mg/mL) [18] and skin (GPY: 3.98 mg/mL; YGPM: 0.73 mg/mL) [67], tuna cardiac arterial bulbs (GEQSN: 1.857 mg/mL; GEGQR: 2.143 mg/mL) [50], seaweed (YLL: 1.61 mg/mL; FYKA: 1.91 mg/mL) [31] and Antarctic krill (VEKT: 0.86 mg/mL; IDSQ: 1.45 mg/mL) [29]. This result indicates that MSP1, MSP2, MSP8, MSP10 and MSP13 have good antioxidant activity similar to that of SOD against $O_{2}^{-}\cdot$ and can be used as $O_{2}^{-}\cdot$ scavengers in living cells.

ABTS^+^· is a single oxygen ion radical with an absorption peak at 734 nm, which can be reduced by antioxidants leading to a decrease in absorbance. The EC_50_ values of MSP1, MSP2 and MSP13 were significantly lower than those of the other fifteen isolated peptides. However, through consulting the literature, it is found that the EC_50_ values of MSP1, MSP2, and MSP13 were significantly higher than those of marine APs from red stingray (*Dasyatis akajei*) cartilages (VPR: 0.15 mg/mL; IEEEQ: 0.18 mg/mL) [11], monkfish skin (GPY: 2.12 mg/mL; YGPM: 0.82 mg/mL) [62], scalloped hammerhead sharks muscle (WDR: 0.34 mg/mL; SAP: 0.19 mg/mL; PYFK: 0.12 mg/mL) [63,64], tuna milt (GHHAAA: 1.12 mg/mL; SMDV: 1.16 mg/mL) [68], tuna roes (GHHAAA: 1.12 mg/mL; SMDV: 1.16 mg/mL) [65] and tuna cardiac arterial bulbs (GEGQR: 1.218 mg/mL; GLN: 1.641 mg/mL) [50].

### 3.3. Structure–Activity Relationship of MSP1, MSP2, MSP7, MSP8, MSP10 and MSP13

Presently, numerous APs derived from a variety of proteins and their bioactivity has been investigated using in vitro and in vivo methods. In general, MW, AA composition and sequence, and spatial structure are generally regarded as critical factors for their activities [61,69,70]. In the study, MSP1, MSP2, MSP7, MSP8, MSP10 and MSP13 belong to tripeptides, tetrapeptides and pentapeptides with MWs of 574.54, 539.49, 417.46, 431.49, 490.47 and 503.54 Da, respectively. Therefore, smaller molecule size is very conducive to the binding of MSP1, MSP2, MSP7, MSP8, MSP10 and MSP13 and targets to play their functions [71,72,73].

The roles of AA composition, especially hydrophobic/aromatic AAs, are often discussed in previous literature [3,12,28]. Hydrophobic/aromatic AAs can facilitate the binding between the peptides and ROS by improving the peptides’ solubility in the reactive solution [21,70]. Ala residue should play an important role in the antioxidant activity of MSP8 (ARW) and MSP13 (YPAGP). Aromatic AAs contain a benzene ring structure, which can provide hydrogen ions to convert ROS into more stable phenoxy radicals and control the peroxide domino effects mediated by ROS [10,20,74]. Sheng et al. reported that Tyr and Phe residues in GEYGFE and Phe residue in IELFPGLP exerted key roles in their antioxidant activities [20]. Therefore, Tyr residue in MSP1 (YDYD), MSP2 (QDYD) and MSP13 (YPAGP) and Trp residue in MSP7 (GRW) and MSP8 (ARW) could positively affect their activity. In addition, Pro residue could improve the flexibility of peptides and act as proton/hydrogen donors to remove ROS directly [24,31,75]. Therefore, Pro residue should be important for the activity of MSP13 (YPAGP).

Hydrophilic AA residues also are necessary for the activity of APs. Acidic (Asp, Glu, Asn and Gln) and basic (Lys and Arg) AA residues have been proven as excellent chelating agents of metal-ions because the excessive electrons in their carboxylic group could improve electrostatic and ionic with metal-ion to play their excellent metal-chelating function [10,76]. Therefore, basic (Arg and Lys) and acidic (Glu and Asp) AA residues were frequently found in APs, such as LKPGN [29], VPR, IEPH, LEEEE and IEEEQ [11], LDEPDPLI and NTDGSTDYGILQINSR [77], PHPR, VRDQY [54] and AEDKKLIQ [78]. Therefore, Asp residue in MSP1 (YDYD), Gln and Asp residues in MSP2 (QDYD), Arg residue in MSP7 (GRW) and MSP8 (ARW) and Asp and Lys residues in MSP10 (DDGGK) must be a great help to their antioxidant ability. Gly residue is often found in collagen peptides with antioxidant activity, such as GFRGTIGLVG, GPAGPAG, GFPSG [13], FTGMD, GFEPY, GFYAA, GIEWA [79], PFGPD, PYGAKG and YGPM [50] because it can maintain the high flexibility of peptide chain and act as a single hydrogen donor to neutralize ROS. Then, Gly residue presented in MSP7 (GRW), MSP10 (DDGGK) and MSP13 (YPAGP) are helpful for their activity.

## 4. Materials and Methods

### 4.1. Materials and Chemical Reagents

Monkfish (*L. litulon*) swim bladders were kindly provided by Zhejiang Hailisheng Group Co., Ltd. (Zhoushan, China). The voucher specimen (No. DC046) was authenticated by Prof. Sheng-long Zhao (Zhejiang Ocean University, Zhoushan, China) and has been deposited in the School of Food and Pharmacy, Zhejiang Ocean University. Acetonitrile of LC grade and trifluoroacetic acid (TFA) were purchased from Thermo Fisher Scientific Co., Ltd. (Shanghai, China). Glutathione (GSH, PHR1359), 2,2-Diphenyl-1-picrylhydrazyl (DPPH, D9132) and 2,2′-Azino-bis(3-ethylbenzothiazoline-6-sulfonic acid) diammonium salt (ABTS, 11557-1G) were bought from Sigma-Aldrich (Shanghai, China) Trading Co., Ltd. Papain, alcalase, trypsin, and pepsin was purchased from Beijing Genthold Biotechnology Co., Ltd. (Beijing, China). Sephadex G-15 were purchased from Shanghai Source Poly Biological Technology Co., Ltd. (Shanghai, China). Peptides of MSP1 to MSP18 (>98%) were synthesized by Shanghai Apeptide Co., Ltd. (Shanghai, China). Human hepatocarcinoma cell lines (HepG2) were purchased from the Shanghai Cell Bank of the Chinese Academy of Sciences.

### 4.2. Preparation of MSBH

#### 4.2.1. Screening of Enzyme Species

The monkfish swim bladders were homogenated and defatted using isopropanol [79]. After that, the defatted powder of monkfish swim bladders was dispersed in a buffer solution to prepare 5% (*w*/*v*) protein slurry and hydrolyzed, respectively using pepsin (pH 2.0, 37 °C), trypsin (pH 7.8, 37 °C), alcalase (pH 9.0, 50 °C), papain (pH 7.0, 55 °C), flavourzyme (pH 7.0, 50 °C) with enzyme dose 2.0%. After 4 h, the monkfish swim bladder hydrolysates were placed in boiling water for 20 min, and the inactivated hydrolysates were centrifuged at 4000× *g* for 25 min. The resulting supernatant was freeze-dried and kept at −20 °C. The monkfish swim bladder hydrolysate produced using papain showed the highest DPPH· scavenging activity.

#### 4.2.2. Optimization of Hydrolysis Conditions of Papain

A single-factor experiment was used to optimize the hydrolysis conditions of papain. Hydrolysis temperature (55, 60, 65, 70, and 75 °C), time (3, 4, 5, 6 and 7 h), pH (6.0, 6.5, 7.0, 7.5 and 8.0) and enzyme dose (1.0, 1.5, 2.0, 2.5 and 3%) were chosen for the following procedure.

According to the above results, the orthogonal experiment was applied to evaluate the effects of hydrolysis conditions (temperature (A), time (B), pH (C) and enzyme dose (D)) on DPPH· scavenging activity of hydrolysates. Three levels (A: 60, 65 and 70 °C; B: 7.0, 7.5 and 8.0; C: 1.5, 2.0 and 2.5%; D: 3, 4 and 5 h) were identified for evaluating the influencing of hydrolysis conditions on DPPH· scavenging rate of hydrolysates. In addition, the monkfish swim bladder hydrolysate produced under the optimized conditions of papain was referred to as MSBH.

### 4.3. Preparation of APs from MSBH

#### 4.3.1. Ultrafiltration of MSBH

MSBH was fractionated using cut-off membranes with MWs of 1, 3.5 and 10 kDa and four peptide fractions of MSBH-I (MW < 1.0 kDa), MSBH-II (1.0 < MW < 3.5 kDa), MSBH-III (3.5 < MW < 10 kDa) and MSBH-IV (MW > 10 kDa) were prepared [80]. MSBH-I showed the highest radical scavenging activity.

#### 4.3.2. Purification of APs from MSBH-I by Chromatography Methods

MSBH-I solutions (5 mL, 50.0 mg/mL) were loaded into the Sephadex G-15 column (2.0 × 120 cm) and eluted using ultrapure water at a flow rate of 0.8 mL/min. The eluent was collected each 2 min, and three fractions (MSBH-Ia, MSBH-Ib and MSBH-Ic) were collected according to the chromatographic diagram at 220 nm.

MSBH-Ib was further isolated using a Zorbax, SB C-18 column (4.6 × 250 mm, 5 µm) in the HPLC system. The Zorbax column was eluted by a linear gradient of acetonitrile (0–50% in 0–30 min) in 0.1% TFA. The eluent at a flow rate of 1.0 mL/min was detected at 214 and 254 nm. Finally, sixteen peaks (P1 to P16) were prepared on the HPLC chromatograms at 214 and 254 nm and freeze-dried.

### 4.4. Identification of Eighteen Isolated APs (MSP1-MSP18)

The AA sequences of eighteen peptides (MSP1-MSP18) from monkfish swim bladders were determined by a 494-protein sequencer of Applied Biosystems (Perkin Elmer Co., Ltd. Foster City, CA, USA). The MWs of eighteen peptides (MSP1-MSP18) were determined by a Q-TOF mass spectrometer with an ESI source (Micromass, Waters, Milford, MA, USA) [81,82].

### 4.5. Antioxidant Activity of MSP1 to MSP18

#### 4.5.1. HO· Scavenging Activity

First, 1.0 mL of 1, 10-phenanthroline solution (1.87 mM) and 2.0 mL of the sample solution were added to a screw-capped tube and mixed. Then, 1.0 mL of a FeSO_4_·7H_2_O solution (1.87 mM) was added to the mixture. The reaction was initiated by adding 1.0 mL of H_2_O_2_ (0.03%, *v*/*v*). After incubating at 37 °C for 60 min, the absorbance of the reaction mixture was measured at 536 nm against a reagent blank. The reaction mixture without any antioxidants was used as the negative control, and a mixture without H_2_O_2_ was used as the blank. The HO· scavenging activity was calculated using the following formula:

HO· scavenging activity (%) = [(A_s_ − A_n_)/(A_b_ − A_n_)] × 100%,

where A_s_, A_n_ and A_b_ are the absorbance values determined at 536 nm of the sample, the negative control and the blank after the reaction, respectively.

#### 4.5.2. DPPH· Scavenging Activity

A total of 2.0 mL of sample solution consisting of distilled water and different concentrations of the analytes was added in cuvettes, and 500 μL of an ethanolic solution of DPPH (0.02%) and 1.0 mL of ethanol were added. A control sample containing the DPPH solution without the sample was also prepared. In the blank, the DPPH solution was substituted with ethanol. The antioxidant activity of the sample was evaluated using the inhibition percentage of the DPPH· with the following equation:

DPPH· scavenging activity (%) = (A_c_ + A_b_ − A_s_)/A_c_ × 100%,

where A_s_ is the absorbance rate of the sample, A_c_ is the control group absorbance and A_b_ is the blank absorbance.

#### 4.5.3. $O_{2}^{-}\cdot$ Scavenging Activity

Superoxide anions were generated in 1 mL of nitrotetrazolium blue chloride (NBT) (2.52 mM), 1 mL of NADH (624 mM) and 1 mL of different sample concentrations. The reaction was initiated by adding 1 mL of phenazine methosulphate (PMS) solution (120 μM) to the reaction mixture. The absorbance was measured at 560 nm against the corresponding blank after incubation for 5 min at 25 °C. The scavenging capacity of the $O_{2}^{-}\cdot$ was calculated using the following equation:

$$O_{2}^{-}\cdot \mathrm{scavenging}\mathrm{activity}(\%)=[(A_{c}-A_{s})/A_{c}]\times 100\%,$$

where A_c_ is the absorbance without the sample and A_s_ is the absorbance with the sample.

#### 4.5.4. ABTS^+^· Scavenging Activity

The ABTS^+^· was generated by mixing ABTS stock solution (7 mM) with potassium persulphate (2.45 mM). The mixture was left in the dark at room temperature for 16 h. The ABTS^+^· solution was diluted in 5 mM phosphate buffered saline (PBS) pH 7.4, to an absorbance of 0.70 ± 0.02 at 734 nm. One milliliter of diluted ABTS^+^· solution was mixed with one milliliter of different concentrations of samples. Ten minutes later, the absorbance was measured at 734 nm against the corresponding blank. The ABTS^+^· scavenging activity of samples was calculated using the following equation:

ABTS^+^· scavenging activity (%) = [(A_c_ − A_s_)/A_c_] × 100%,

where A_c_ was the absorbance without the sample and A_s_ was the absorbance with the sample.

#### 4.5.5. Determination of Reducing Power

Generally, 2.0 mL of each sample dissolved in distilled water was mixed with 2.5 mL of 1% aqueous potassium hexacyanoferrate [K_3_Fe(CN)_6_] solution. After 30 min incubation at 50 °C, 1.5 mL of 10% trichloroacetic acid was added. Finally, 2.0 mL of the upper layer was mixed with 2.0 mL of distilled water and 0.5 mL of 0.1% aqueous FeCl_3_ and the absorbance was recorded at 700 nm. The higher absorbance of the reaction mixture indicated the stronger reducing power.

#### 4.5.6. Lipid Peroxidation Inhibition Assay

Briefly, a sample (5.0 mg) was dissolved in 10 mL of 50 mM PBS (pH 7.0) and added to 0.13 mL of a solution of linoleic acid and 10 mL of 99.5% ethanol. Then, the total volume was adjusted to 25 mL with deionized water. The mixture was incubated in a conical flask with a screw cap at 40 °C in a dark room, and the degree of oxidation was evaluated by measuring ferric thiocyanate values. The reaction solution (100 μL) incubated in the linoleic acid model system was mixed with 4.7 mL of 75% ethanol, 0.1 mL of 30% ammonium thiocyanate, and 0.1 mL of 20 mM ferrous chloride solution in 3.5% HCl. After 3 min, the thiocyanate value was measured at 500 nm following color development with FeCl_2_ and thiocyanate at different intervals during the incubation period at 40 °C. The higher absorbance of the solution means lower lipid peroxidation inhibition capacity.

#### 4.5.7. Protective Functions on Plasmid DNA of MSP1, MSP2, MSP7, MSP8, MSP10 and MSP13

The protective functions of MSP1, MSP2, MSP7, MSP8, MSP10 and MSP13 on plasmid DNA (pBR322) were determined using the previous method [83]. In short, peptide (MSP1, MSP2, MSP7, MSP8, MSP10 or MSP13, respectively) were added to the test tubes containing FeSO_4_ (2 µL, 1.0 mM), pBR322 (1 µL, 0.5 µg) and H_2_O_2_ (2 µL, 1.0 mM). A total of 15 µL of the manufactured reaction solution was incubated at 37 °C. After 30 min, 2 µL of loading buffer was added to the solution. Then, the solution was subsequently electrophoresed on 1% agarose gel containing 0.5 µg/mL EtBr at 60 V for 50 min. Finally, the DNA in the agarose gel was photographed and recorded under ultraviolet light.

#### 4.5.8. Cytoprotection of MSP1, MSP2, MSP7, MSP8, MSP10 and MSP13 on H_2_O_2_-Damaged HepG2 Cells

H_2_O_2_ (300 µM) was used for establishing the oxidative damage model of HepG2 cells according to the previous methods [84,85,86]. Briefly, the HepG2 cells were incubated in a 96-well plate for 24 h. The supernatant in a 96-well plate was aspirated, and peptide solution (100 µL, 100.0 µM) was added into the sample groups and incubated for 8 h. After removing peptides, H_2_O_2_ was added to the sample, GSH and model groups. After 24 h, the 96-wells were rinsed twice with PBS and used MTT method to determine the cell viability:

Cell viability = (OD_sample_/OD_control_) × 100%.

### 4.6. Stability of MSP1, MSP2, MSP7, MSP8, MSP10 and MSP13

The stability of MSP1, MSP2, MSP7, MSP8, MSP10 and MSP13 was determined according to the previous method with a light modification [87,88,89]. The $O_{2}^{-}\cdot$ scavenging activity (%) of MSP1, MSP2, MSP7, MSP8, MSP10 and MSP13 at 5.0 mg/mL were measured to evaluate their stability.

The thermostability of MSP1, MSP2, MSP7, MSP8, MSP10 and MSP13 was analyzed in a water bath for 2 h with temperatures of 25, 37, 60, 80 or 100 °C, respectively.

The pH values (3, 5, 7, 9 and 11) were set to evaluate the acid and alkali stability properties of MSP1, MSP2, MSP7, MSP8, MSP10 and MSP13 at 25 °C, and the incubating time with different pH solutions was set to 2 h.

Two-stage simulated GI digestion model (2 h of pepsin digestion followed by 2 h of trypsin digestion) was designed to evaluate the influence of simulated GI digestion on the stability of MSP1, MSP2, MSP7, MSP8, MSP10 and MSP13.

### 4.7. Statistical Analysis

All the data are expressed as the mean ± SD (*n* = 3). The experimental data were analyzed by an ANOVA test using SPSS 19.0. Significant differences were determined by Duncan’s multiple range test (*p* < 0.05, 0.01, and 0.001).

## 5. Conclusions

In conclusion, the conditions of papain for hydrolyzing the protein of monkfish (*L. litulon*) swim bladders were optimized as hydrolysis temperature 65 °C, pH 7.5, enzyme dose 2.5% and time 5 h through single factor and orthogonal experiments, and eighteen APs (MSP1 to MSP18) were purified from the monkfish swim bladder hydrolysate and identified as YDYD, QDYD, AGPAS, GPGPHGPSGP, GPK, HRE, GRW, ARW, GPTE, DDGGK, IGPAS, AKPAT, YPAGP, DPT, FPGPT, GPGPT, GPT and DPAGP, respectively. In general, YDYD, ARW and DDGGK exhibited high ability on radical scavenging, lipid peroxidation inhibition, Ferric reducing antioxidant power, and protective function on oxidation-damaged Plasmid DNA and HepG2 cells. The antioxidant activity of eighteen isolated peptides (MSP1 to MSP18) was highly stable under high temperatures, but remarkably influenced by different pH and simulated GI digestion. In brief, the present finding provides a good perspective for monkfish processing byproducts-swim bladders as the high-quality biological resources to produce BPs, and the generated peptides could serve as antioxidative ingredients applied in health-promoting products. Moreover, the antioxidant mechanism of peptides (MSP1, MSP2, MSP7, MSP8, MSP10 and MSP13) and the therapeutic effects of these peptides on HepG2 cells and mice after oxidative damage will be systematically researched in our follow-up study.

## Figures and Tables

**Table 1:** Results of the orthogonal experiment for optimizing the hydrolysis conditions of papain.

No	Factors				DPPH· Scavenging Activity (%)
	A Temperature (°C)	B pH	C Enzyme Dose (%)	D Time (h)	
1	1 (60)	1 (7.0)	1 (1.5)	1 (3)	38.58 ± 1.39
2	1 (60)	2 (7.5)	2 (2.0)	2 (4)	43.33 ± 0.92
3	1 (60)	3 (8.0)	3 (2.5)	3 (5)	43.00 ± 0.38
4	2 (65)	1 (7.0)	2 (2.0)	3 (5)	43.47 ± 0.45
5	2 (65)	2 (7.5)	3 (2.5)	1 (3)	44.63 ± 0.65
6	2 (65)	3 (8.0)	1 (1.5)	2 (4)	41.59 ± 1.05
7	3 (70)	1 (7.0)	3 (2.5)	2 (4)	44.08 ± 0.87
8	3 (70)	2 (7.5)	1 (1.5)	3 (5)	42.67 ± 2.52
9	3 (70)	3 (8.0)	2 (2.0)	1 (3)	38.67 ± 0.52
K1	124.91	126.13	122.84	121.88	
K2	129.69	130.63	125.47	129.00	
K3	125.42	123.26	131.71	129.14	
k1	41.64	42.04	40.95	40.63	
k2	43.23	43.54	41.82	43.00	
k3	41.81	41.09	43.90	43.05	
Best level	A_2_	B_2_	C_3_	D_3_	
R	1.59	2.45	2.95	2.42	
R order	C > B > D > A				

**Table 2:** Retention time (RT, min), amino acid (AA) sequences and molecular weights (MWs) of eighteen isolated APs (MSP1-MSP18) from MSBH.

Peaks	RT (min)	AA Sequence	Theoretical/Observed MW (Da)
P1	6.08	MSP1: Tyr-Asp-Tyr-Asp (YDYD)	574.54/574.54
		MSP2: Gln-Asp-Tyr-Asp (QDYD)	539.49/539.49
P2	7.14	MSP3: Ala-Gly-Pro-Ala-Ser (AGPAS)	401.41/401.41
P3	8.22	MSP4: Gly-Pro-Gly-Pro-His-Gly- Pro-Ser-Gly-Pro (GPGPHGPSGP)	858.90/858.89
P4	8.30	MSP5: Gly-Pro-Lys (GPK)	300.35/300.35
P5	8.93	MSP6: His-Arg-Glu (HRE)	440.45/440.45
P6	9.02	MSP7: Gly-Arg-Trp (GRW)	417.46/417.46
P7	9.87	MSP8: Ala-Arg-Trp (ARW)	431.49/431.49
P8	10.89	MSP9: Gly-Pro-Thr-Glu (GPTE)	402.40/402.40
P9	10.91	MSP 10: Asp-Asp-Gly-Gly-Lys (DDGGK)	490.47/490.47
P10	12.60	MSP11: Ile-Gly-Pro-Ala-Ser (IGPAS)	443.49/443.49
		MSP12: Ala-Lys-Pro-Ala-Thr (AKPAT)	486.56/486.56
P11	13.92	MSP13: Tyr-Pro-Ala-Gly-Pro (YPAGP)	503.55/503.54
P12	14.93	MSP14:Asp-Pro-Thr (DPT)	331.32/331.32
P13	15.02	MSP15: Phe-Pro-Gly-Pro-Thr (FPGPT)	517.57/517.57
P14	17.18	MSP16: Gly-Pro-Gly-Pro-Thr (GPGPT)	427.45/427.45
P15	17.99	MSP17: Gly-Pro-Thr (GPT)	273.29/273.29
P16	20.50	MSP18: Asp-Pro-Ala-Gly-Pro (DPAGP)	455.46/455.46

**Table 3:** EC_50_ values of eighteen isolated APs (MSP1-MSP18) on HO·, DPPH·, $O_{2}^{-}\cdot$ and ABTS^+^·.

Peptides	EC_50_ (mg/mL)			
	DPPH·	HO·	$O_{2}^{-}\cdot$	ABTS^+^·
MSP1	2.824 ± 0.019 ^a^	0.150 ± 0.060 ^a^	0.126 ± 0.0005 ^a^	3.197 ± 0.036 ^a^
MSP2	2.993 ± 0.054 ^a^	0.177 ± 0.035 ^a,b^	0.112 ± 0.0028 ^b^	2.337 ± 0.016 ^b^
MSP3	8.667 ± 0.023 ^b^	0.475 ± 0.0103 ^c^	0.166 ± 0.0017 ^c^	6.693 ± 0.030 ^c^
MSP4	7.703 ± 0.091 ^c^	0.244 ± 0.0035 ^d^	0.397 ± 0.0008 ^d^	7.613 ± 0.133 ^d^
MSP5	10.947 ± 0.031 ^d^	0.652 ± 0.0132 ^e^	0.239 ± 0.0026 ^e^	6.641 ± 0.043 ^c^
MSP6	5.231 ± 0.083 ^e^	0.349 ± 0.0037 ^f,g^	0.188 ± 0.0026 ^f^	5.729 ± 0.025 ^e,i^
MSP7	1.053 ± 0.003 ^f^	0.201 ± 0.013 ^b^	0.245 ± 0.0021 ^g^	6.188 ± 0.084 ^f^
MSP8	0.773 ± 0.003 ^g^	0.183 ± 0.0016 ^a,b^	0.127 ± 0.0002 ^a^	4.503 ± 0.040 ^g^
MSP9	9.420 ± 0.109 ^h^	0.493 ± 0.0042 ^c^	0.173 ± 0.0020 ^h^	8.034 ± 0.124 ^h^
MSP10	10.417 ± 0.110 ^i^	0.329 ± 0.004 ^f,g,h^	0.128 ± 0.0018 ^a^	6.004 ± 0.087 ^f,i^
MSP11	8.195 ± 0.271 ^j^	0.366 ± 0.004 ^f,g,h^	0.335 ± 0.0027 ^i^	8.054 ± 0.366 ^h^
MSP12	13.96 ± 0.284 ^k^	0.482 ± 0.0309 ^c^	0.353 ± 0.0006 ^j^	8.695 ± 0.200 ^j^
MSP13	2.821 ± 0.012 ^a^	0.190 ± 0.010 ^b^	0.107 ± 0.0002 ^k^	3.839 ± 0.102 ^k^
MSP14	4.450 ± 0.005 ^l^	0.353 ± 0.0067 ^f,g,h^	0.116 ± 0.0002 ^b^	5.411 ± 0.028 ^e^
MSP15	11.013 ± 0.042 ^d^	0.306 ± 0.0025 ^g^	0.505 ± 0.0058 ^l^	9.058 ± 0.082 ^l^
MSP16	13.99 ± 0.046 ^k^	0.312 ± 0.0065 ^f,g^	0.208 ± 0.0080 ^m^	10.89 ± 0.322 ^m^
MSP17	10.413 ± 0.006 ^i^	0.439 ± 0.0069 ^i^	0.185 ± 0.0014 ^f^	12.387 ± 0.670 ^n^
MSP18	7.119 ± 0.092 ^m^	0.332 ± 0.035 ^f,g,h^	0.166 ± 0.0020 ^c^	5.621 ± 0.169 ^e^

All data are presented as the mean ± SD of triplicate results. ^a–n^ Values with the same letters indicate no significant difference in each column (*p* > 0.05).

**Table 4:** Declined percentage of $O_{2}^{-}\cdot$ scavenging activity of MSP1, MSP2, MSP7, MSP8, MSP10 and MSP13 subjected to different pH treatments.

Peptides	Declined Percentage (%)				
	pH 3	pH 5	pH 7	pH 9	pH 11
MSP1	−21.77	−21.10	0.00	−4.97	−35.26
MSP2	−36.14	−33.16	0.00	−2.29	−73.07
MSP7	−18.68	−16.43	0.00	−5.78	−69.19
MSP8	−19.97	−19.47	0.00	−3.85	−83.05
MSP10	−28.91	−24.92	−5.13	−7.06	0
MSP13	−26.03	−21.19	−0.15	0	−26.03

**Table 5:** Declined percentage of $O_{2}^{-}\cdot$ scavenging activity of MSP1, MSP2, MSP7, MSP8, MSP10 and MSP13 subjected to simulated GI digestion treatments.

	Declined Percentage (%)		
	Untreated	Treated with Pepsin	Treated with Pepsin and Trypsin
MSP1	0.00	−0.84	−4.10
MSP2	0.00	−15.13	−47.59
MSP7	0.00	−24.34	−49.19
MSP8	0.00	−24.93	−28.62
MSP10	0.00	−21.98	−53.33
MSP13	0.00	−28.02	−35.83

**Table 6:** Declined percentage of $O_{2}^{-}\cdot$ scavenging activity of MSP1, MSP2, MSP7, MSP8, MSP10 and MSP13 subjected to different thermal treatments.

Peptides	Declined Percentage (%)				
	25 °C	37 °C	60 °C	80 °C	100 °C
MSP1	0.00	−1.48	−0.82	−1.15	−0.82
MSP2	0.00	−1.00	−1.50	−3.17	−3.84
MSP7	0.00	−1.94	−0.52	−0.70	−0.53
MSP8	0.00	1.02	0.34	−0.17	−1.70
MSP10	0.00	−0.87	−1.58	−2.16	−2.60
MSP13	0.00	0.00	0.00	0.61	−0.46

## Data Availability

Data are contained within the article.

## Abbreviations

DPPH: 2,2-diphenyl-1-picrylhydrazyl; HO·, hydroxyl radical; $O_{2}^{-}\cdot$, superoxide anion radical; ABTS, 2,2′-Azinobis-(3-ethylbenzthiazoline-6-sulphonate; MTT, 3-(4,5)-dimethylthiahiazo (-z-y1)-3,5-di- phenytetrazoliumromide; BP, bioactive peptide; AA, amino acid; MW, molecular weight; ROS, reactive oxygen species; NCD, chronic non-communicable diseases; WHO, World Health Organization; AP, antioxidant peptide; AMPK, AMP-activated protein kinase; TG, triglyceride; TC, total cholesterol; Nrf2, NF-E2-related factor 2; ARE, antioxidant response element; Keap1, Kelch Like ECH-Associated Protein 1; NF-κB, nuclear factor kappa-light-chain-enhancer of activated B cells; PI3K, phosphoinositide 3-kinases; MafK, Maf bZIP transcription factor K; NAFLD, non-alcoholic fatty liver disease; NLRP3, NLR family pyrin domain containing 3; MSBH, protein hydrolysate of monkfish swim bladders; MSP, peptide of monkfish swim bladders; FRAP, Ferric reducing antioxidant power; GSH, glutathione; SC, supercoiled; LIN, linear; OC, open circular; GI, gastrointestinal; Q-TOF, Quadrupole time-of-flight.
